# Mental Health Among Medical Students During COVID-19: A Systematic Review and Meta-Analysis

**DOI:** 10.3389/fpsyg.2022.846789

**Published:** 2022-05-10

**Authors:** Qingwen Jia, Yi Qu, Huiyuan Sun, Huisheng Huo, Hongxia Yin, Dianping You

**Affiliations:** ^1^Graduate School, Hebei Medical University, Shijiazhuang, China; ^2^Editorial Department of Nursing Practice and Research, Children’s Hospital of Hebei Province, Shijiazhuang, China; ^3^Department of Scientific Research, Children’s Hospital of Hebei Province, Shijiazhuang, China; ^4^Party and Government Integrated Office, Children’s Hospital of Hebei Province, Shijiazhuang, China

**Keywords:** COVID-19, medical students, depression, anxiety, meta-analysis

## Abstract

**Background:**

The mental health of medical students is an issue worthy of attention, especially during COVID-19. Many studies have shown that depression and anxiety are the main problems faced by medical students. To assess the pooled prevalence of depression and anxiety among medical students worldwide, we conducted this meta-analysis.

**Methods:**

According to PRISMA, we used a computerized strategy to search studies in EMBASE, PubMed, PsycArticles, Web of Science, and China Biology Medicine disc. The pooled prevalence of depression and anxiety was calculated by a random-effects model. Heterogeneity was explored by subgroup analysis. Sensitivity analysis and publication bias were also carried out in this meta-analysis.

**Results:**

Of 1316 studies, 41 studies were selected based on 36608 medical students. The pooled depression prevalence was 37.9% (95% CI: 30.7–45.4%), and pooled anxiety prevalence was 33.7% (95% CI: 26.8–41.1%). The prevalence of depression and anxiety among medical students varied by gender, country, and continent.

**Conclusion:**

The data reported that the prevalence of depression and anxiety among medical students during COVID-19 was relatively higher than those of the general population and the healthcare workers. The impact of COVID-19 on medical students and how to protect the mental health of medical students are needed to determine through further research.

**Systematic Review Registration:**

[https://www.crd.york.ac.uk/prospero/display_record.php?ID=CRD42021274015], identifier [CRD42021274015].

## Introduction

College students were considered to be a sensitive and special group, and their mental health seemed to be more troubled, as their vulnerability is exacerbated by their inability to adapt to the new environment of universities, higher education plans, and the insufficient identification and utilization of social resources (Acharya et al., 2018). Many studies had investigated the mental health problems of college students and explored the related influencing factors. They suggested that college students’ mental health was affected by academic education, psychological elasticity, stress level, and other factors (Torres et al., 2017; Acharya et al., 2018; Alqudah et al., 2021; Alyoubi et al., 2021).

In December 2019, unexplained pneumonia suddenly broke out, which had swept the globe in a short time (Galea et al., 2020). With numerous infected people appearing every day, the government took lockdown measures to control and prevent the serious epidemic (Pierce et al., 2020; Tran et al., 2020; Basheti et al., 2021; Fancourt et al., 2021). Some studies had reported that mental health was related to COVID-19. A cross-sectional study conducted in China shows that Chinese people’s anxiety, depression, and drinking levels are more significant than before, and their mental health status decreases during the outbreak of COVID-19 (Ahmed et al., 2020). Some studies have shown that anxiety and depression are common mental health problems faced by college students during COVID-19, and they are at a high risk level (Naser et al., 2020); similarly, research results show that college students are vulnerable to psychological problems during the pandemic of COVID-19 and isolation and distance learning has a significant impact on students’ anxiety levels (Alqudah et al., 2021); In addition to COVID-19 patients and medical staff, college students are another group of people who are particularly prone to mental health disorders during the pandemic. Even if not during the outbreak of COVID-19, college students are experiencing considerable anxiety and depression due to academic pressure (Deng et al., 2021). Some related meta-analyses also confirmed this, the prevalence of depression and anxiety among college students increased during the COVID-19 (Deng et al., 2021; Guo et al., 2021).

Compared with other higher education, medical education was regarded as one of the training programs with the highest academic and emotional requirements out of any profession (Azad et al., 2017; Quek et al., 2019; Zeng et al., 2019). This demand and pressure caused a negative impact on the medical students’ mental health (Basudan et al., 2017; Shao et al., 2020). School closures, online teaching, and the inability to complete hospital internships had changed the inherent training model of medical students (Abbasi et al., 2020; Byrnes et al., 2020; Farooq et al., 2020; Soled et al., 2020; Bilgi et al., 2021). The study of medical students of bezmialem vakif University shows that many students are deeply affected by the epidemic process, no matter how long they have studied in medical school, one of the main reasons is the interruption of educational activities (Bilgi et al., 2021). The challenges of COVID-19 to global medical students’ mental health were unknown, especially the pooled prevalence of depression and anxiety, although some researchers were concerned about medical students’ mental health during this special period (Liu et al., 2020; Nakhostin-Ansari et al., 2020; Gupta et al., 2021). However, available studies had varied widely in terms of countries, assessments, and sample sizes, and in addition, there had been considerable variations in the reported prevalence of depression and anxiety. Therefore, for future researchers to quickly grasp the depression and anxiety of medical students during the epidemic, to facilitate their further research, this meta-analysis was conducted.

## Methods

### Data Sources and Search Strategy

This systematic review and meta-analysis had already been registered on PROSPERO before review initiation (CRD42021274015). We searched five databases (EMBASE, PubMed, PsycArticles, Web of Science, and China Biology Medicine disc) to identify the studies, and the final retrieval time of the literature was August 18, 2021. A search strategy consisting of three separate parts was applied to each database (“anxiety” OR “depression”) AND (“medical student*” OR “students, medical*”) AND (“COVID-19” OR “pneumonia” OR “Coronavirus” OR “SARS-COV-2”), and the publication time was limited to 2019-2021. The reference lists of relevant articles were searched for additional eligible papers (Supplementary Material 1).

### Selection Criteria

Literature inclusion criteria: (1) The sample population consisting of students from medical colleges or medical-related majors; (2) Validated instrument was used to screen depression or anxiety and explicit cutoff value was given in the article; (3) Research conducted during COVID-19; (4) Published articles in English. Exclusion criteria were: (1) Qualitative studies, oral presentations, letter or non-original research; (2) Medical students with mental illness were not excluded; (3) Lack of useful information or the data needed.

### Study Selection

After duplicate publications were excluded, irrelevant literature was further excluded through titles and abstracts. Then appraised the remaining articles according to inclusion and exclusion criteria of literature formulated in advance by reading the full texts. Reference lists of the selected articles were checked to identify further articles. The whole process was completed by two researchers independently, and disagreements were resolved through discussion, or a third arbitrator, if necessary.

### Data Collection

Two researchers independently reviewed the full text of eligible studies and extracted the following data by using the predefined standardized form: The first author, year of publication, country, study period, study design, sample size, female ratio, assessment tools, and the event of depression or anxiety. For longitudinal studies, data collected during COVID-19 were included. Disagreements were resolved by consensus.

### Quality Assessment

AHRQ (Agency for Healthcare Research and Quality criteria) was used to evaluate the methodological quality of the literature included in our meta-analysis, which is suitable for cross-sectional studies (Zhang et al., 2021b). There are 11 questions in total, those who meet the requirements will get 1 point. After scoring each item, according to the total score, each study quality was assessed as follows: low-quality = 0–3; moderate-quality = 4–7; high-quality = 8–11.

### Analysis

We utilized software R (“meta” package) to perform meta-analytic calculations. To ensure that the prevalence proportions conform to the normal distribution, we converted the data through PRAW (untransformed), PLN (log transformation), PLOGIT (logit transformation), PAS (arcsine transformation), and PFT (Freeman–Tukey double arcsine transformation) (Barendregt et al., 2013; Luo et al., 2021). Based on the high expected heterogeneity between studies, the random-effects model was used in our meta-analyses. We calculated the pooled prevalence of depression and anxiety, and its corresponding 95% confidence interval (CI). According to the recommendations of the Cochrane Handbook, heterogeneity was estimated by Cochran’s Q test (*p* < 0.10) and the I^2^ statistic: the cutoff value of 75% indicates high heterogeneity. The source of heterogeneity was explored through subgroup analysis, we conducted additional subgroup analysis by countries, assessment tools, gender, and continents. The stability and reliability of pooled prevalence were evaluated by sensitivity analysis (Liu et al., 2021). Egger test of bias was used to assess the publication bias (Egger et al., 1997).

## Results

### Literature Screening

**FIGURE 1:**
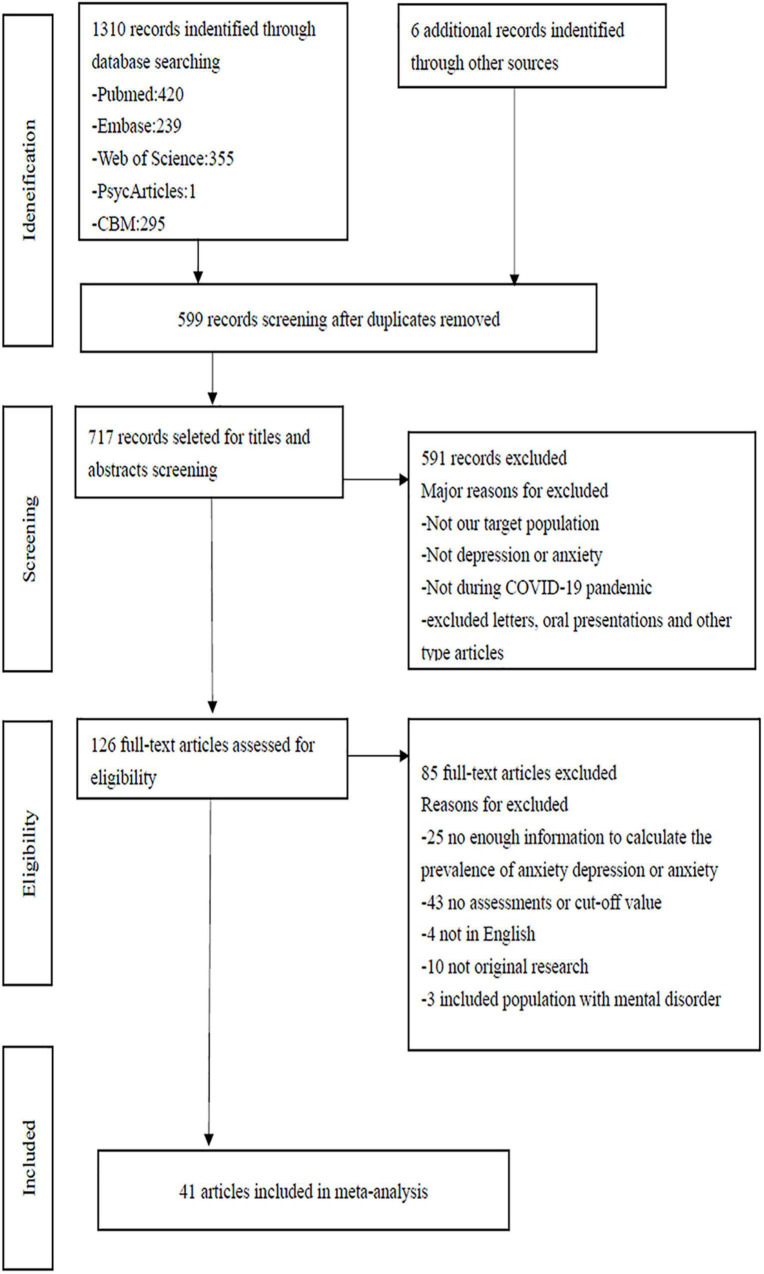
Flow chart of study selection.

A total of 1310 articles were identified from the electronic database; 6 additional papers were found through a references list check. First, 465 duplicate literatures were excluded, two researchers independently evaluated the remaining 717 articles through title and abstract, irrelevant literature was further excluded. And then appraised the remaining articles according to inclusion and exclusion criteria of literature formulated in advance, 591 articles that did not meet the inclusion criteria were further excluded. The remaining 126 articles were evaluated through reading the full text to determine whether they were included in the meta-analysis. Reference lists of the selected articles were checked to identify further articles. The whole process was completed by two researchers independently, and disagreements were resolved through discussion, or a third arbitrator, if necessary. Ultimately, 41 studies were included in meta-analysis (Figure 1).

### Study Characteristics

Table 1 shows the overall characteristics of the 41 included studies (Elhadi et al., 2020; Jindal et al., 2020; Liu et al., 2020; Medeiros et al., 2020; Muhammad Alfareed Zafar et al., 2020; Nadeem et al., 2020; Nakhostin-Ansari et al., 2020; Nihmath Nisha et al., 2020; Sartorao Filho Carlos et al., 2020; Sun et al., 2020; Adhikari et al., 2021; Banstola et al., 2021; Basheti et al., 2021; Bilgi et al., 2021; Gao et al., 2021; Guo et al., 2021; Gupta et al., 2021; Halperin et al., 2021; Kalkan Uğurlu et al., 2021; Kaplan Serin and Doğan, 2021; Keskin, 2021; Kuman Tunçel et al., 2021; Li et al., 2021; Mekhemar et al., 2021; Meng et al., 2021; Nishimura et al., 2021; Patelarou et al., 2021; Pavan et al., 2021; Pelaccia et al., 2021; Perissotto et al., 2021; Saeed and Javed, 2021; Safa et al., 2021; Song et al., 2021; Xiao et al., 2021; Xie et al., 2021; Yadav et al., 2021; Yang et al., 2021; Yin et al., 2021; Zhang et al., 2021a; Zheng et al., 2021; Zhu et al., 2021). On the whole, 40 cross-sectional studies and 1 longitudinal study were included in our study. We included the two surveys of the longitudinal study, as they were conducted during the pandemic (Gao et al., 2021). Included studies came from 15 countries, including China, Turkey, the United States, Nepal, Brazil, India, and other countries. The included studies were conducted from January 2020 to February 2021. The minimum sample size of the included study is more than 100 and the maximum sample size is 6348. One of the studies was a transnational study, and the sample population came from Albania (*n* = 197), Greece (*n* = 348), and Spain (*n* = 242) (Patelarou et al., 2021). Of the 41 included studies, 38 studies reported anxiety prevalence and 31 studies reported depression prevalence. In total, 36608 participants were included. One study included 195 participants, 6 of whom only participated in the depression survey and did not complete the anxiety-related survey (Gupta et al., 2021).

**TABLE 1:** Characteristics of 41 included studies.

Author, Year	Country	Survey time (2020)	Study design	Sample size (N)	Female (%)	Assessment		Quality of study
						Depression	Anxiety	
Adhikari et al., 2021	Nepal	Aug–Sep	CS	223	39.5	PHQ-9	/	Moderate
Banstola et al., 2021	Nepal	Jan–Feb *	CS	144	100	/	BAI-21	Moderate
Basheti et al., 2021	Jordan	Jul	CS	450	67.1	HADS	HADS	Moderate
Bilgi et al., 2021	Turkey	Jun	CS	178	71.3	PHQ-9	GAD-7	Moderate
Elhadi et al., 2020	Libya	Apr–May	CS	2430	79	PHQ-9	GAD-7	Moderate
Gao et al., 2021	China	Jun–Oct	LS	702	71.3	DASS-21	DASS-21	Moderate
Guo et al., 2021	America	Jun–Aug	CS	852	/	/	GAD-7	Moderate
Gupta et al., 2021	America	Apr	CS	195	/	PHQ-9	GAD-7	Moderate
Halperin et al., 2021	America	Apr	CS	1428	66.7	PHQ-9	GAD-7	Moderate
Jindal et al., 2020	India	May	CS	762	/	/	GAD-7	Moderate
Kalkan Uğurlu et al., 2021	Turkey	May	CS	411	79.3	DASS-42	DASS-42	Moderate
Kaplan Serin and Doğan, 2021	America	Jun	CS	344	70.9	/	GAD-7	Moderate
Keskin, 2021	Turkey	Sep	CS	259	60.2	DASS-42	DASS-42	Moderate
Kuman Tunçel et al., 2021	Turkey	Apr–May	CS	3105	56.7	/	BAI-21	Moderate
Li et al., 2021	China	Mar	CS	6348	90.4	PHQ-9	GAD-7	Moderate
Liu et al., 2020	China	Feb–Apr	CS	217	58.5	PHQ-9	GAD-7	Moderate
Medeiros et al., 2020	Brazil	May	CS	113	77	HADS	HADS	Moderate
Mekhemar et al., 2021	Germany	Jul–Jan**	CS	211	73.5	DASS-21	DASS-21	Moderate
Meng et al., 2021	China	Feb	CS	1624	/	PHQ-9	GAD-7	High
Muhammad Alfareed Zafar et al., 2020	Pakistan	Mar–Apr	CS	323	/	SDS	SAS	Moderate
Nadeem et al., 2020	Pakistan	Jun	CS	281	69.3	/	GAD-7	Low
Nakhostin-Ansari et al., 2020	Iran	Apr	CS	323	52.3	BDI-II	BAI -21	Moderate
Nihmath Nisha et al., 2020	India	Apr–Jun	CS	359	49.6	CES-D	GAD-7	Moderate
Nishimura et al., 2021	Japan	Apr	CS	473	34	PHQ-9	GAD-7	Moderate
Patelarou et al., 2021	Greece	Apr–May	CS	348	84.8	PHQ-9	/	Moderate
	Spain			242	85.5			
	Albania			197	80.2			
Pavan et al., 2021	India	Aug	CS	233	41.3	/	GAD-7	Moderate
Pelaccia et al., 2021	France	May	CS	1165	65.2	/	STAI-A	Moderate
Perissotto et al., 2021	Brazil	Mar–Jun	CS	347	65.9	HADS	HADS	Moderate
Saeed and Javed, 2021	Pakistan	Jun–Aug	CS	234	47.4	PHQ-9	GAD-7	Moderate
Safa et al., 2021	Bangladesh	Apr–May	CS	425	62.4	HADS	HADS	Moderate
Sartorao Filho Carlos et al., 2020	Brazil	May	CS	340	73.8	PHQ-9	GAD-7	Moderate
Song et al., 2021	China	Feb	CS	435	/	SDS	SAS	Moderate
Sun et al., 2020	China	Feb–Mar	CS	474	84.8	/	SAS	Moderate
Xiao et al., 2021	China	Feb	CS	933	70.1	PHQ-9	GAD-7	Moderate
Xie et al., 2021	China	Feb	CS	1026	63.6	SDS	/	Moderate
Yadav et al., 2021	Nepal	Jun	CS	409	83.1	PHQ-9	GAD-7	Moderate
Yang et al., 2021	China	Apr–May	CS	212	88.2	/	SAS	Moderate
Yin et al., 2021	China	Feb	CS	5982	60	PHQ-9	GAD-7	Moderate
Zhang et al., 2021a	China	Apr	CS	1041	52.4	DASS-21	DASS-21	Moderate
Zheng et al., 2021	China	Dec	CS	468	/	PHQ-9	GAD-7	Moderate
Zhu et al., 2021	China	Mar–Apr	CS	342	86.8	PHQ-9	GAD-7	Moderate

“/” mean Not reported. Study design: LS, longitudinal study; CS, cross-sectional study. Assessment of depression: PHQ-9, Patient Health Questionnaire-9; SDS, Self-Rating Depression Scale; BDI-II, Beck Depression Inventory-II; CES-D, Center for Epidemiology Studies for Depression. Assessment of anxiety: BAI-21, Beck Anxiety Inventory 21-item; GAD-7, Generalized Anxiety Disorder 7-item Scale; STAI-A, State-Trait Anxiety Inventory; SAS, Self-Rating Anxiety Scale. Assessment of anxiety and depression: DASS-21, Depression Anxiety and Stress Scale-21; DASS-42, Depression Anxiety and Stress Scale-42; HADS, Hospital Anxiety and Depression Scale. *Data collection from January to February- 2021. **Data collection from July 2020 to January 2021.

### Quality of Study

According to the methodological quality evaluation of the literature by two researchers, forty studies were of moderate quality, one of high quality (Meng et al., 2021), and one of low quality (Nadeem et al., 2020). Our meta-analysis will not include low quality studies (Supplementary Material 2).

### Prevalence of Depression and Anxiety

**FIGURE 2:**
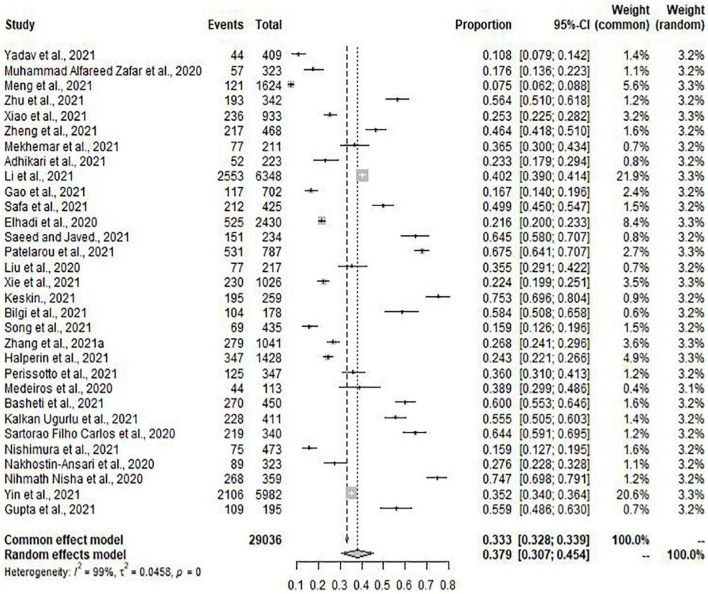
Forest plot for depression.

**FIGURE 3:**
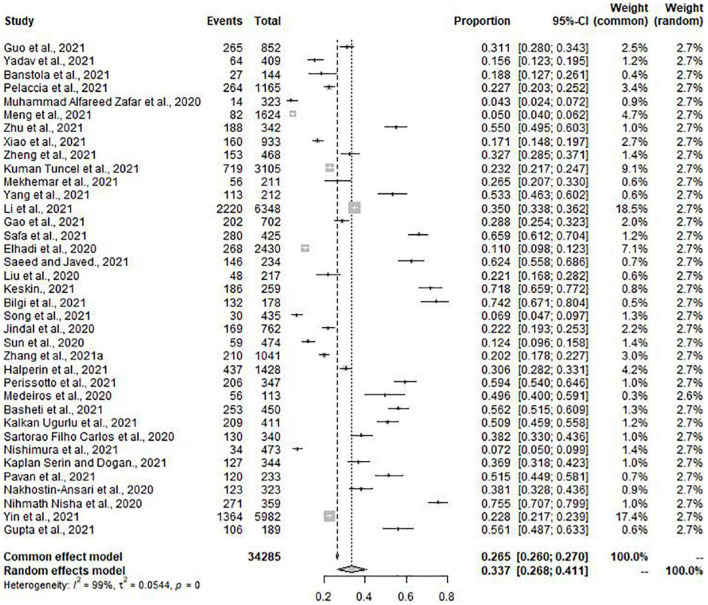
Forest plot for anxiety.

Meta-analysis included 31 of the total studies with depression (*n* = 29036), the pooled prevalence of depression was 37.9% (95% CI: 30.7–45.4%) with high heterogeneity (*I*^2^ = 99%, *p* = 0.00) (Figure 2). Of the 41 studies, 37 studies (*n* = 34285) with the condition of anxiety were conducted to meta-analysis. The pooled prevalence of anxiety was 33.7% (95% CI: 26.8–41.1%) with high heterogeneity (*I*^2^ = 99%, *p* = 0.00) (Figure 3).

### Subgroup Analysis

According to subgroup analysis of assessment instruments, the lowest pooled prevalence of anxiety was 16.1% (95% CI: 2.0–39.9%) used SAS with high heterogeneity (*I*^2^ = 99%, *p* < 0.01). The pooled prevalence of depression used SDS was 18.8% (95% CI: 14.9–23.0%) with high heterogeneity (*I*^2^ = 79%, *p* < 0.01) (Table 2).

**TABLE 2:** Subgroup analysis of assessment.

Depression					Anxiety				

Assessment	No. of Studies	P (%) mean (95%CI)	I^2^	P value	Assessment	No. of Studies	P (%) mean (95%CI)	I^2^	P value
PHQ-9	17	37.1% (27.4%–47.4%)	99%	0.00	GAD-7	20	33.5% (24.1%–43.6%)	99%	0.00
SDS	3	18.8% (14.9%–23.0%)	79%	< 0.01	SAS	4	16.1% (2.0% –39.9%)	99%	< 0.01
BDI-II	1	27.6% (22.8%–32.8%)	/	/	BAI-21	3	26.4% (16.2%–38.1%)	94%	< 0.01
CES-D	1	74.7% (69.8%–79.1%)	/	/	STAI-A	1	22.7% (20.3%–25.2%)	/	/
DASS-21	3	26.0% (15.7%–37.9%)	95%	< 0.01	DASS-21	3	24.9% (19.6%–30.6%)	89%	< 0.01
HADS	4	46.4% (35.6%–57.4%)	94%	< 0.01	HADS	4	58.5% (52.2%–64.6%)	78%	< 0.01
DASS-42	2	65.6% (45.3%–83.3%)	96%	< 0.01	DASS-42	2	61.5% (40.4%–80.6%)	97%	< 0.01

**FIGURE 4:**
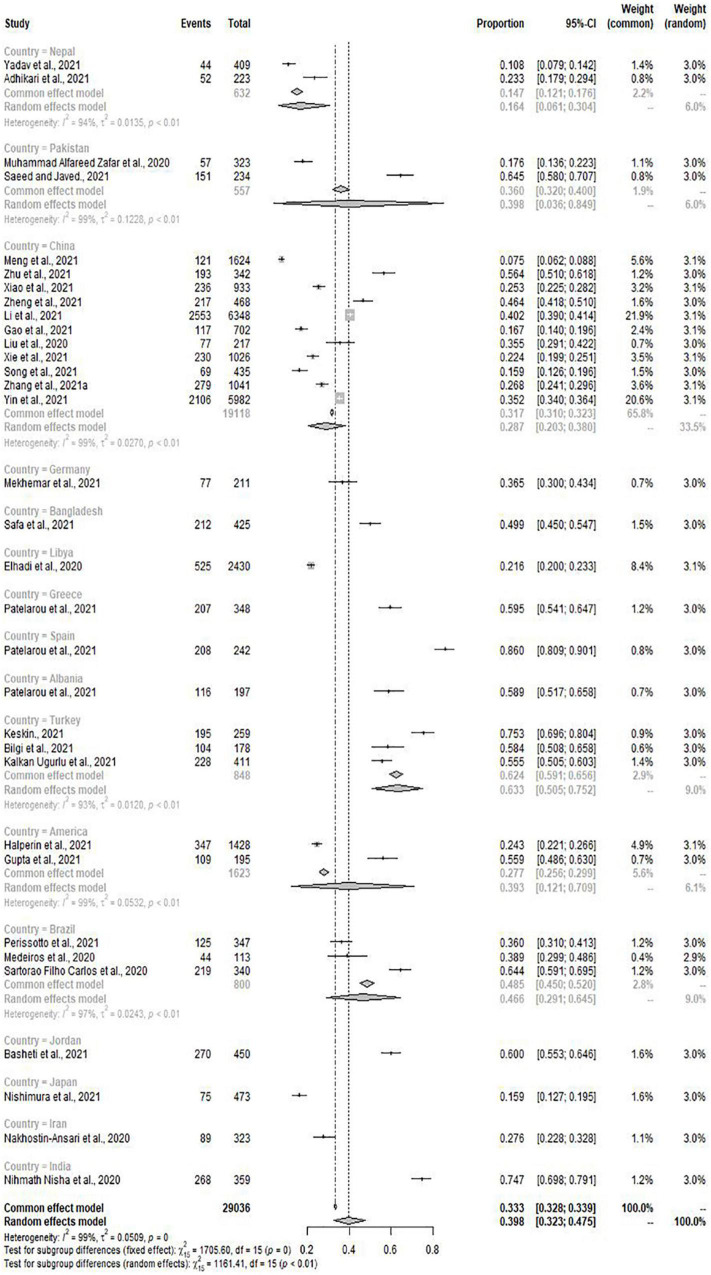
Subgroup analysis by countries for depression.

**FIGURE 5:**
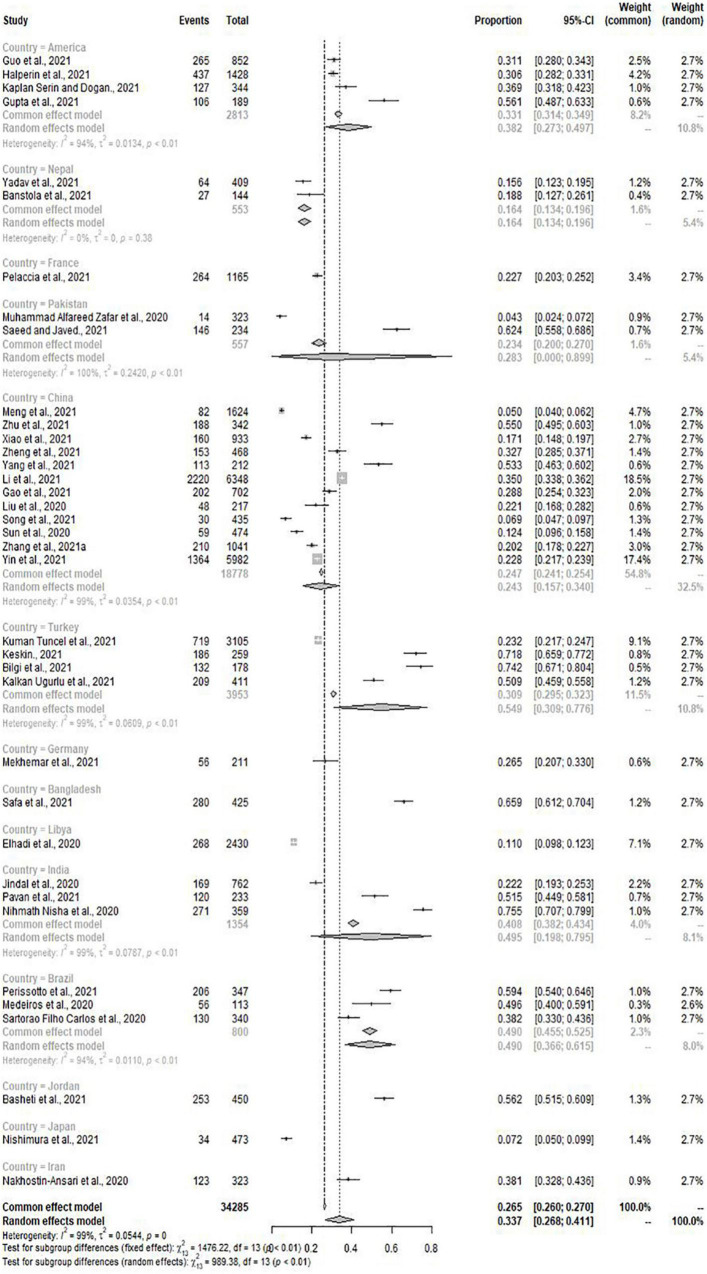
Subgroup analysis by countries for anxiety.

When classified according to countries, the prevalence of depression and anxiety in Japan was significantly lower than in other countries (15.9%, 95% CI: 12.7–19.5%; 7.2%, 95% CI: 5.0–9.9%). Interestingly, the prevalence of depression in Spanish medical students was the highest (86.0%, 95% CI: 80.9–90.1%), and Bangladesh had the highest prevalence of anxiety among many countries (65.9%, 95% CI: 61.2–70.4%) (Figures 4, 5). Then, we conducted subgroup analysis according to the geographical location of the countries, and found that the prevalence of depression was the highest (52.3%, 95% CI: 22.9–80.8%) but the prevalence of anxiety was relatively low (23.2%, 95% CI: 21.0–25.5%) in Europe. At the same time, we found that the pooled prevalence of anxiety in female was higher than that in male (33.8%, 95% CI: 23.6–45.9%; 28.4%, 95% CI:19.2–40.0%), the pooled prevalence of depression also showed this characteristic (36.7%, 95% CI: 27.3–46.6%; 32.2%, 95% CI:22.4–42.8%) (Table 3).

**TABLE 3:** Subgroup analysis based on gender and region.

Subgroup		Depression				Anxiety			
		No. of Studies	P (%) mean (95%CI)	I^2^	P value	No. of Studies	P (%) mean (95%CI)	I^2^	P value
Gender	Male	13	32.2% (22.4%–42.8%)	96%	< 0.01	14	28.4% (19.2%–40.0%)	98%	< 0.01
	Female	13	36.7% (27.3%–46.6%)	98%	< 0.01	15	33.8% (23.6%–45.9%)	99%	< 0.01
Region	Africa	1	21.6% (20.0%–23.3%)	/	/	1	11.0% (9.8%–12.3%)	/	/
	Asia	23	36.2% (27.6%–45.3%)	99%	0.00	27	33.2% (24.4%–42.6%)	99%	0.00
	Europe	2	52.3% (22.9%–80.8%)	98%	< 0.01	2	23.2% (21.0%–25.5%)	34%	0.22
	North America	2	39.3% (12.1%–70.9%)	99%	< 0.01	4	38.2% (27.3%–49.7%)	94%	< 0.01
	South America	3	46.6% (29.1%–64.5%)	97%	< 0.01	3	49.0% (36.6%–61.5%)	94%	< 0.01

### Sensitivity Analysis and Publication Bias

**FIGURE 6:**
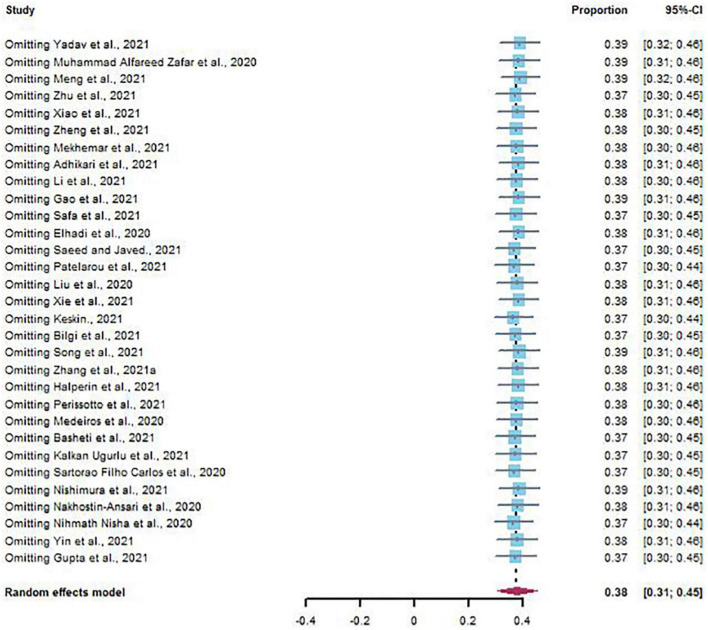
Sensitivity analysis of depression.

**FIGURE 7:**
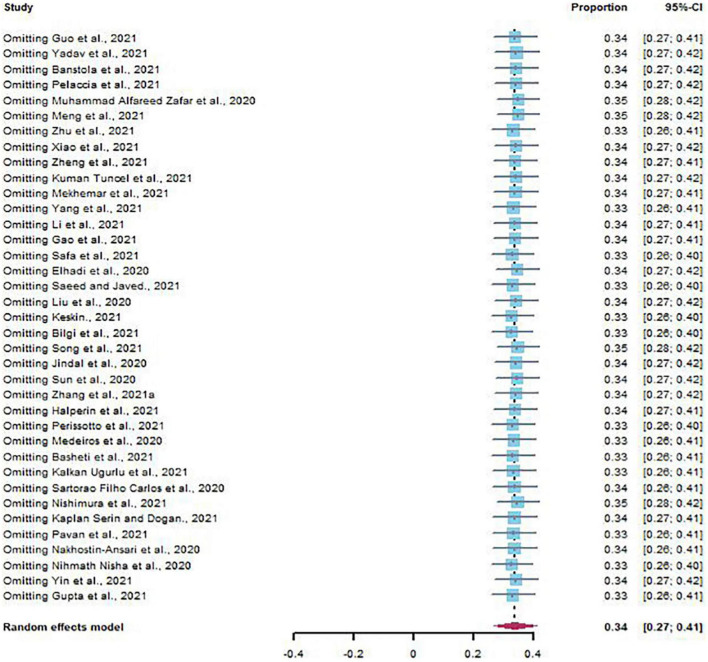
Sensitivity analysis of anxiety.

**FIGURE 8:**
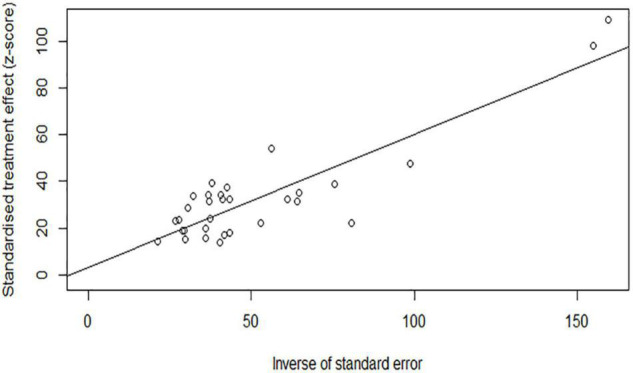
Egger’s regression test for depression.

**FIGURE 9:**
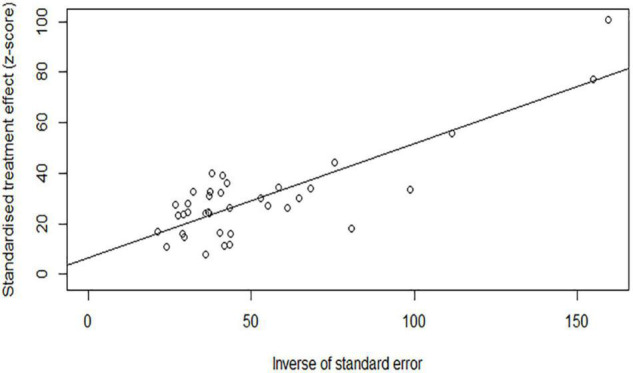
Egger’s regression test for anxiety.

The results of sensitivity analysis showed that there was no significant change in the prevalence of depression and anxiety (Figures 6, 7). Similarly, Egger’s regression test showed that there was no publication bias on depression and anxiety (*p* = 0.3742, *p* = 0.0528) (Figures 8, 9).

## Discussion

We conducted this systematic review and meta-analysis to determine the prevalence of depression and anxiety in medical students over the world during the COVID-19 pandemic. In our study, we find that the pooled prevalence of depression and anxiety among medical students was 37.9%, 95% CI: 30.7–45.4%; 33.7%, 95% CI: 26.8–41.1%, more prominent relative to the general population and healthcare workers (Pappa et al., 2020; Castaldelli-Maia et al., 2021; Cénat et al., 2021; Raoofi et al., 2021; Sahebi et al., 2021; Santabárbara et al., 2021). This seemed to be consistent with the prevalence of anxiety reported by Quek et al. (2019) (33.8%, 95% CI: 29.2–38.7%). However, we found that the research they included had a heavy period, from 1998 to 2019, while the research we included was more concentrated. We inferred that an excessive period might have an impact on the pooled prevalence of anxiety. Another study reported that the prevalence of anxiety among medical students during the epidemic was 28%, which was lower than our conclusion (Lasheras et al., 2020). We found that their latest literature search time was August 26, 2020, although the epidemic situation had been partially controlled at that time, its influence still existed. Even in China, medical students deferred enrollment until October 1, 2020. Repeated signs of the epidemic situation and the fear of returning to school, the impact of these factors on the mental health among medical students needed to be considered. In addition, we also found that the prevalence of depression among medical students was relatively high during the COVID-19 compared with before (Puthran et al., 2016; Rotenstein et al., 2016).

Interestingly, when we performed a subgroup analysis based on the sample source countries, we found that the pooled prevalence of depression and anxiety among Chinese medical students was not prominent (28.7%, 95% CI: 20.3–38.0%; 24.3%, 95% CI: 15.7–34.0%). We further divided countries according to continents and conducted subgroup analysis. It was noted that the prevalence of depression was the highest in Europe (52.3%, 95% CI: 22.9–80.8%) and relatively low in Asia (36.2%, 95% CI: 27.6–45.3%). In terms of anxiety prevalence, Europe is at a low level (23.2%, 95% CI: 21.0–25.5%), and South America has the highest anxiety prevalence (49.0%, 95% CI: 36.6–61.5%). Research showed that rumors about the epidemic affected the mental health of citizens (Ahmed et al., 2020; Xiong et al., 2020), and the mental health of the population was also related to the nationwide epidemic control (Jiang, 2020). Limited outdoor activities, increased new cases and fear of the possibility to be infected were identified to impact mental health (Jiang, 2020; Wang et al., 2020c; Kaplan Serin and Doğan, 2021). In particular, the closure of schools and online teaching had brought unprecedented challenges to the education of medical students (Bilgi et al., 2021), and the effects of social distance and self-isolation might make students feel more vulnerable and lonelier, increasing depression and anxiety symptoms (Huang et al., 2020). Similarly, in some countries, medical students were limited to a clinical internship during the epidemic. Sudden changes impacted the traditional training mode of medical students (Abbasi et al., 2020; Keskin and Özkan, 2021). The impact of COVID-19 on psychological and mental health can be reduced by timely updating the relevant accurate information such as the number of new epidemic cases and the route of transmission (Wang et al., 2020a). Benefit from the rapid and effective measures taken by the Chinese government, the epidemic was quickly and effectively controlled and the public panic was reduced (Lau et al., 2020; Pan et al., 2020; Li et al., 2021).

Finally, we found that the prevalence of depression and anxiety in female medical students was higher than that in males, and some previous studies have reported a similar situation (Puthran et al., 2016; Quek et al., 2019; Alyoubi et al., 2021; Luo et al., 2021). Females seemed to be more vulnerable to mental health problems than males (Baxter et al., 2013; Qiu et al., 2020; Xiong et al., 2020; Kalkan Uğurlu et al., 2021), we speculated that it was related to females unique physiological and psychological factors: females are more likely to articulate their worries and emotions (Basudan et al., 2017; Lin et al., 2021). When we performed subgroup analysis according to the assessment tools, we found that different depression and anxiety assessment tools also brought different prevalence rates of depression and anxiety. Due to the diversity of sample sources and the high heterogeneity of subgroup analysis, we could not infer the impact of different assessment tools on the prevalence of depression and anxiety among medical students.

COVID-19 has a huge impact on mental health. An American study showed that the prevalence of depressive symptoms was more than 3 times higher during COVID-19 compared with before (Ettman et al., 2020). Economic turmoil, home quarantine and the uncertainty of COVID-19 cases had brought great stress to the people, accompanied by an increase in the level of anxiety and depression during the COVID-19 pandemic (Shehata et al., 2021). Studies have shown that strict government policies slow down the spread of COVID-19, but such interventions disrupt daily life and lead to adverse mental health outcomes, especially strict blockade measures and home confinement with unknown duration. Epidemiological monitoring and targeted intervention should be implemented in time to prevent further mental health problems (Rossi et al., 2020; Wang et al., 2020b; Ding et al., 2021). Personal exposure to COVID-19 is an important risk factor for increased anxiety and depressive symptoms during pandemic (Ding et al., 2021). The prevalence of anxiety was more significant in people who had infected with COVID-19 or knew someone who had experienced illness (Shabahang et al., 2021). In addition, less exercise and lack of social support can also lead to more anxiety and depression symptoms (Kong et al., 2020; Shah et al., 2021).

Although study had shown that the harm caused by COVID-19 pandemic to the overall mental health of the population will improve over time (Gallagher et al., 2021), epidemics and other health emergencies may lead to harmful and long-term psychosocial consequences, which cannot be ignored (Dong and Bouey, 2020). Even if the epidemic ends, its negative socio-economic consequences, such as work difficulties, may also have an adverse impact on the mental health of the population (Rossi et al., 2020). Without intervention and appropriate health and social policies, mental health problems will have serious adverse consequences. The government plays an important role in reducing the prevalence of anxiety and depression during COVID-19. The government’s decisive and rapid epidemic prevention measures can help to reduce the further spread of the COVID-19 and protect the mental health of the public (Wang et al., 2020b; Zhang et al., 2021c). In addition, family companionship can reduce anxiety and depression levels (Shah et al., 2021).

## Conclusion

In conclusion, this systematic review and meta-analysis reported a relatively high prevalence of depression and anxiety. The prevalence of depression and anxiety was 37.9%, 33.7%, higher than that of the general population and healthcare workers. The prevalence varied in different countries. Researchers can further explore the differences and influencing factors of mental health among medical students with different cultural backgrounds.

### Limitations and Strengths

In conclusion, this systematic review and meta-analysis reported a relatively high prevalence of depression and anxiety. For its high heterogeneity, we tried to use extensive subgroup analysis to reveal the source of heterogeneity. According to the results of subgroup analysis, we found that the combined prevalence was not reversed, indicating that our results remained relatively stable. Secondly, only studies published in English were eligible to be included in the meta-analysis, which limits the estimation of prevalence to a certain extent. In addition, most studies were descriptive, the association between COVID-19 and medical students’ depression and anxiety may not imply a causal relation. Moreover, the prevalence of the included studies was estimated by self-report, and the differences in individual emotional expressions are also factors that need further consideration. Then, due to the small number of studies in some countries, although we combine countries into continents for subgroup analysis, the number of studies in Africa is still small, and future researchers should pay attention to this problem. However, we have to say that our meta-analysis includes a sizeable sample size (n = 36608). Despite some limitations, the findings still have some key significance. Importantly, they support other researchers to grasp the prevalence of depression and anxiety in medical students during COVID-19, to make corresponding psychological intervention measures.

## Data Availability Statement

The original contributions presented in the study are included in the article/Supplementary Material, further inquiries can be directed to the corresponding author.

## Author Contributions

QJ conducted the data analyses and wrote the manuscript. HH and HY conducted the literature search. YQ and HS conducted the study quality assessment. DY conducted the supervision, review, and editing. All authors approved the final manuscript.

## Conflict of Interest

The authors declare that the research was conducted in the absence of any commercial or financial relationships that could be construed as a potential conflict of interest.

## Publisher’s Note

All claims expressed in this article are solely those of the authors and do not necessarily represent those of their affiliated organizations, or those of the publisher, the editors and the reviewers. Any product that may be evaluated in this article, or claim that may be made by its manufacturer, is not guaranteed or endorsed by the publisher.
