# Supplementary material for: Mental Health Among Medical Students During COVID-19: A Systematic Review and Meta-Analysis
Source: Front Psychol. 2022 May 10;13:846789. doi: 10.3389/fpsyg.2022.846789 (PMC9127415; doi:10.3389/fpsyg.2022.846789)
Supplement: Supplementary file 1 [file Data_Sheet_1.docx]

**S1 Search strategy**

**1. Search strategy in PubMed**

#1 (("covid 19"[All Fields] OR "covid 19"[MeSH Terms] OR "covid 19 vaccines"[All Fields] OR "covid 19 vaccines"[MeSH Terms] OR "covid 19 serotherapy"[All Fields] OR "covid 19 serotherapy"[Supplementary Concept] OR "covid 19 nucleic acid testing"[All Fields] OR "covid 19 nucleic acid testing"[MeSH Terms] OR "covid 19 serological testing"[All Fields] OR "covid 19 serological testing"[MeSH Terms] OR "covid 19 testing"[All Fields] OR "covid 19 testing"[MeSH Terms] OR "sars cov 2"[All Fields] OR "sars cov 2"[MeSH Terms] OR "severe acute respiratory syndrome coronavirus 2"[All Fields] OR "ncov"[All Fields] OR "2019 ncov"[All Fields] OR (("coronavirus"[MeSH Terms] OR "coronavirus"[All Fields] OR "cov"[All Fields]) AND 2019/11/01:3000/12/31[Date - Publication]) OR ("betacoronavirus"[MeSH Terms] OR "betacoronavirus"[All Fields] OR "betacoronaviruses"[All Fields]) OR ("coronavirus"[MeSH Terms] OR "coronavirus"[All Fields] OR "coronaviruses"[All Fields]) OR ("coronavirus infections"[MeSH Terms] OR ("coronavirus"[All Fields] AND "infections"[All Fields]) OR "coronavirus infections"[All Fields]) OR ("pneumonia"[MeSH Terms] OR "pneumonia"[All Fields] OR "pneumoniae"[All Fields] OR "pneumonias"[All Fields] OR "pneumoniae s"[All Fields]) OR ("pneumonia, viral"[MeSH Terms] OR ("pneumonia"[All Fields] AND "viral"[All Fields]) OR "viral pneumonia"[All Fields] OR ("pneumonia"[All Fields] AND "viral"[All Fields]) OR "pneumonia viral"[All Fields]) OR ("sars cov 2"[MeSH Terms] OR "sars cov 2"[All Fields] OR "sars cov 2"[All Fields]) OR ("pneumonia, bacterial"[MeSH Terms] OR ("pneumonia"[All Fields] AND "bacterial"[All Fields]) OR "bacterial pneumonia"[All Fields] OR ("pneumonia"[All Fields] AND "bacterial"[All Fields]) OR "pneumonia bacterial"[All Fields]))

#2((("medic"[All Fields] OR "medical"[All Fields] OR "medicalization"[MeSH Terms] OR "medicalization"[All Fields] OR "medicalizations"[All Fields] OR "medicalize"[All Fields] OR "medicalized"[All Fields] OR "medicalizes"[All Fields] OR "medicalizing"[All Fields] OR "medically"[All Fields] OR "medicals"[All Fields] OR "medicated"[All Fields] OR "medication s"[All Fields] OR "medics"[All Fields] OR "pharmaceutical preparations"[MeSH Terms] OR ("pharmaceutical"[All Fields] AND "preparations"[All Fields]) OR "pharmaceutical preparations"[All Fields] OR "medication"[All Fields] OR "medications"[All Fields]) AND "student*"[All Fields]) OR ("students, medical"[MeSH Terms] OR ("students"[All Fields] AND "medical"[All Fields]) OR "medical students"[All Fields] OR ("medical"[All Fields] AND "student"[All Fields]) OR "medical student"[All Fields]) OR (("student s"[All Fields] OR "students"[MeSH Terms] OR "students"[All Fields] OR "student"[All Fields] OR "students s"[All Fields]) AND "medical*"[All Fields]) OR ("students, medical"[MeSH Terms] OR ("students"[All Fields] AND "medical"[All Fields]) OR "medical students"[All Fields] OR ("students"[All Fields] AND "medical"[All Fields]) OR "students medical"[All Fields]))

#3("anxiety"[MeSH Terms] OR "anxiety"[All Fields] OR "anxieties"[All Fields] OR "anxiety s"[All Fields] OR ("depressed"[All Fields] OR "depression"[MeSH Terms] OR "depression"[All Fields] OR "depressions"[All Fields] OR "depression s"[All Fields] OR "depressive disorder"[MeSH Terms] OR ("depressive"[All Fields] AND "disorder"[All Fields]) OR "depressive disorder"[All Fields] OR "depressivity"[All Fields] OR "depressive"[All Fields] OR "depressively"[All Fields] OR "depressiveness"[All Fields] OR "depressives"[All Fields]) OR (("depressed"[All Fields] OR "depression"[MeSH Terms] OR "depression"[All Fields] OR "depressions"[All Fields] OR "depression s"[All Fields] OR "depressive disorder"[MeSH Terms] OR ("depressive"[All Fields] AND "disorder"[All Fields]) OR "depressive disorder"[All Fields] OR "depressivity"[All Fields] OR "depressive"[All Fields] OR "depressively"[All Fields] OR "depressiveness"[All Fields] OR "depressives"[All Fields]) AND "disorder*"[All Fields]) OR ("depressive disorder"[MeSH Terms] OR ("depressive"[All Fields] AND "disorder"[All Fields]) OR "depressive disorder"[All Fields])))

#4 Filters from 2019/1/1 - 2021/8/18

#5 #1 AND #2 AND #3 AND #4

**2. Search strategy in Web of Science**

#1 Topic = anxiety OR depression OR depressive disorder* OR depressive disorder

#2 Topic = medical student* OR medical student OR Students, Medical* OR Students, Medical

#3 Topic = COVID-19 OR Betacoronavirus OR Coronavirus OR Coronavirus Infections OR Pneumonia OR Pneumonia, Viral OR SARS-CoV-2 OR Pneumonia, Bacterial OR novel coronavirus

#4 PY=2019-2021

#5 #1 AND #2 AND #3 AND #4

**3. Quick Search strategy in Embase**

#1. 'anxiety'/exp OR anxiety

#2. 'anxiety disorder'

#3. 'anxiety assessment'

#4. 'depression'

#5. 'depression assessment'

#6. #1 OR #2 OR #3 OR #4 OR #5

#7. 'medical students'

#8. 'health student'

#9. 'medical student'

#10. 'paramedical student'

#11. 'nursing student'

#12. 'dental student'

#13. 'pharmacy student'

#14. 'premedical student'

#15. 'public health student'

#16. 'students psychology'

#17. #7 OR #8 OR #9 OR #10 OR #11 OR #12 OR #13 OR #14 OR #15 OR #16

#18. #6 AND #17

#19. 'coronavirus disease 2019'

#20. 'sars-cov-2 vaccine'

#21. #19 OR #20

#22. #18 AND #21

**4. Search strategy in Basic Search of PsycArticles**

| **#** | **Query** | **Limiters/Expanders** | **Last Run Via** | **Results** |
| --- | --- | --- | --- | --- |
| S1 | anxiety or depression | Limiters - Linked Full Text | Interface - EBSCOhost Research Databases | 19,010 |
|  |  | Expanders - Apply equivalent subjects | Search Screen - Basic Search |  |
|  |  | Search modes - Find all my search terms | Database - APA PsycArticles |  |
| S2 | students | Limiters - Linked Full Text | Interface - EBSCOhost Research Databases | 37,695 |
|  |  | Expanders - Apply equivalent subjects | Search Screen - Basic Search |  |
|  |  | Search modes - Find all my search terms | Database - APA PsycArticles |  |
| S3 | medical | Limiters - Linked Full Text | Interface - EBSCOhost Research Databases | 16,506 |
|  |  | Expanders - Apply equivalent subjects | Search Screen - Basic Search |  |
|  |  | Search modes - Find all my search terms | Database - APA PsycArticles |  |
| S4 | S2 AND S3 | Limiters - Linked Full Text | Interface - EBSCOhost Research Databases | 1,459 |
|  |  | Expanders - Apply equivalent subjects | Search Screen - Basic Search |  |
|  |  | Search modes - Find all my search terms | Database - APA PsycArticles |  |
| S5 | covid-19 or coronavirus or 2019-ncov or sars-cov-2 or cov-19 | Limiters - Linked Full Text | Interface - EBSCOhost Research Databases | 677 |
|  |  | Expanders - Apply equivalent subjects | Search Screen - Basic Search |  |
|  |  | Search modes - Find all my search terms | Database - APA PsycArticles |  |
| S6 | S1 AND S4 AND S5 | Limiters - Linked Full Text | Interface - EBSCOhost Research Databases | 1 |
|  |  | Expanders - Apply equivalent subjects | Search Screen - Basic Search |  |
|  |  | Search modes - Find all my search terms | Database - APA PsycArticles |  |

**5. Search strategy in CBM (China Biology Medicine disc)**

1) "anxiety"

2) "depression"

3) (#2) OR (#1)

4) "students"

5) "medical"

6) (#4) AND (#5)

7) "COVID-19"

8) "Betacoronavirus"

9) "Coronavirus"

10) "Coronavirus Infections"

11) " Pneumonia"

12) "Pneumonia, Viral"

13) "SARS-CoV-2"

14) "Pneumonia, Bacterial"

15) "novel coronavirus"

16) (#7) OR (#8) OR (#9) OR (#10) OR (#11) OR (#12) OR (#13) OR (#14) OR (#15)

17) (#3) AND (#6) AND (#16)

18) (#3) AND (#6) AND (#16) AND 2019-2021[time]
