# Supplementary material for: Mental Health Among Medical Students During COVID-19: A Systematic Review and Meta-Analysis
Source: Front Psychol. 2022 May 10;13:846789. doi: 10.3389/fpsyg.2022.846789 (PMC9127415; doi:10.3389/fpsyg.2022.846789)
Supplement: Supplementary file 2 [file Data_Sheet_2.docx]

**S2 Methodological quality rating of studies included**

| **Study** | **Define the source of information (survey)** | **List inclusion and exclusion criteria for exposed and unexposed subjects or refer to previous publications** | **Indicate time Period used for identify inpatients** | **Indicate whether or not subjects were consecutive if not population-based** | **Indicate if evaluators of subjective components of study were masked to other aspects of the status of the participants** | **Describe any assessments undertaken for quality assurance purposes** | **Explain any patient exclusions from analysis** | **Describe how confounding was assessed and/or controlled** | **If applicable, explain how missing data were handled in the analysis** | **Summarize patient response rates and completeness of data collection** | **Clarify what follow-up, if any, was expected and the percentage of patients for which incomplete data or follow-up was obtained** |
| --- | --- | --- | --- | --- | --- | --- | --- | --- | --- | --- | --- |
| Guo et al. 2021 | 1 | 0 | 1 | 0 | 0 | 1 | 0 | 0 | 0 | 1 | 0 |
| Yadav et al. 2021 | 1 | 1 | 1 | 0 | 0 | 1 | 0 | 1 | 0 | 0 | 0 |
| Banstola et al. 2021 | 1 | 1 | 1 | 1 | 0 | 1 | 0 | 0 | 0 | 1 | 0 |
| Pelaccia et al. 2021 | 1 | 1 | 1 | 1 | 0 | 1 | 1 | 0 | 0 | 1 | 0 |
| Muhammad Alfareed Zafar et al. 2020 | 1 | 1 | 1 | 1 | 0 | 1 | 0 | 0 | 0 | 1 | 0 |
| Meng et al. 2021 | 1 | 1 | 1 | 1 | 0 | 1 | 1 | 1 | 0 | 1 | 0 |
| Zhu et al. 2021 | 1 | 0 | 1 | 0 | 0 | 1 | 0 | 0 | 0 | 1 | 0 |
| Xiao et al. 2020 | 1 | 0 | 1 | 1 | 0 | 1 | 0 | 0 | 0 | 1 | 0 |
| Zheng et al. 2021 | 1 | 0 | 1 | 0 | 0 | 1 | 0 | 1 | 0 | 0 | 0 |
| Kuman Tunçel et al. 2021 | 1 | 1 | 1 | 1 | 0 | 1 | 0 | 0 | 0 | 0 | 0 |
| Mekhemar et al. 2021 | 1 | 0 | 1 | 1 | 0 | 1 | 0 | 0 | 0 | 0 | 0 |
| Adhikari et al. 2021 | 1 | 0 | 1 | 0 | 0 | 1 | 0 | 0 | 0 | 1 | 0 |
| Yang et al. 2021 | 1 | 1 | 1 | 1 | 0 | 1 | 1 | 0 | 0 | 1 | 0 |
| Li et al. 2021 | 1 | 1 | 1 | 0 | 0 | 1 | 0 | 0 | 0 | 0 | 0 |
| Gao et al. 2021 | 1 | 0 | 1 | 1 | 0 | 1 | 1 | 0 | 0 | 1 | 0 |
| Safa et al. 2021 | 1 | 1 | 1 | 1 | 0 | 1 | 0 | 0 | 0 | 0 | 0 |
| Elhadi  et al. 2020 | 1 | 1 | 1 | 0 | 0 | 1 | 0 | 1 | 0 | 1 | 0 |
| Saeed et al. 2021 | 1 | 1 | 1 | 0 | 0 | 1 | 0 | 0 | 0 | 0 | 0 |
| Patelarou et al. 2021 | 1 | 1 | 1 | 0 | 0 | 1 | 0 | 0 | 0 | 0 | 0 |
| Liu et al. 2020 | 1 | 0 | 1 | 0 | 0 | 1 | 0 | 0 | 0 | 1 | 0 |
| Xie et al. 2021 | 1 | 1 | 1 | 0 | 0 | 1 | 0 | 0 | 0 | 1 | 0 |
| Keskin 2021 | 1 | 0 | 1 | 1 | 0 | 1 | 0 | 0 | 0 | 0 | 0 |
| Bilgi et al. 2021 | 1 | 0 | 1 | 1 | 0 | 1 | 0 | 0 | 0 | 0 | 0 |
| Song et al. 2021 | 1 | 0 | 1 | 0 | 0 | 1 | 0 | 1 | 0 | 1 | 0 |
| Jindal et al. 2020 | 1 | 1 | 1 | 1 | 0 | 1 | 0 | 0 | 0 | 0 | 0 |
| Sun et al. 2020 | 1 | 1 | 1 | 0 | 0 | 1 | 0 | 0 | 0 | 1 | 0 |
| Zhang et al. 2021 | 1 | 1 | 1 | 1 | 0 | 1 | 0 | 1 | 0 | 0 | 0 |
| Halperin et al. 2021 | 1 | 1 | 1 | 1 | 0 | 1 | 0 | 0 | 0 | 1 | 0 |
| Perissotto et al. 2021 | 1 | 0 | 1 | 1 | 0 | 1 | 0 | 0 | 0 | 1 | 0 |
| Medeiros et al. 2020 | 1 | 0 | 1 | 0 | 0 | 1 | 0 | 0 | 0 | 1 | 0 |
| Basheti et al. 2021 | 1 | 1 | 1 | 1 | 0 | 1 | 0 | 0 | 0 | 0 | 0 |
| Kalkan Uğurlu et al. 2021 | 1 | 0 | 1 | 1 | 0 | 1 | 0 | 0 | 0 | 1 | 0 |
| Sartorao Filho Carlos et al. 2020 | 1 | 1 | 1 | 1 | 0 | 1 | 0 | 0 | 0 | 1 | 0 |
| Nishimura et al. 2021 | 1 | 0 | 1 | 1 | 0 | 1 | 0 | 0 | 0 | 1 | 0 |
| Kaplan Serin et al, 2021 | 1 | 1 | 1 | 1 | 0 | 1 | 0 | 0 | 0 | 1 | 0 |
| Pavan et al. 2021 | 1 | 1 | 1 | 1 | 0 | 1 | 1 | 0 | 0 | 1 | 0 |
| Nakhostin-Ansari et al. 2020 | 1 | 0 | 1 | 1 | 0 | 1 | 0 | 0 | 0 | 1 | 0 |
| Nihmath Nisha et al. 2020 | 1 | 0 | 1 | 0 | 0 | 1 | 0 | 0 | 0 | 1 | 0 |
| Nadeem et al. 2020 | 1 | 0 | 1 | 0 | 0 | 1 | 0 | 0 | 0 | 0 | 0 |
| Yin et al. 2021 | 1 | 1 | 1 | 0 | 0 | 1 | 0 | 0 | 0 | 0 | 0 |
| Gupta et al. 2021 | 1 | 1 | 1 | 1 | 0 | 1 | 0 | 0 | 1 | 1 | 0 |

*Note: 1:1 score , 0:0 score*
